# Effect of NaCl and Na_2_SO_4_ on the biodecolourization of K-2BP by *Halomonas* sp. GYW

**DOI:** 10.1080/13102818.2014.901677

**Published:** 2014-04-30

**Authors:** Jing Lian, Zhifang Xu, Jianbo Guo, Lin Yue, Yankai Guo, Chenxiao Zhang, Jingliang Yang

**Affiliations:** ^a^ School of Environmental Science and Engineering, Hebei University of Science and Technology, Shijiazhuang, P.R. China

**Keywords:** *Halomonas* sp. GYW, sodium sulphate, azo dye, oxidation–reduction potential (ORP), Gibbs function

## Abstract

In this paper, the effect of NaCl and Na_2_SO_4_ on the biodecolourization of reactive brilliant red K-2BP by a *Halomonas* sp. GYW (EF188281) was investigated in details. The decolourisation efficiency and the oxidation–reduction potential (ORP) change were explored during the decolourization process. The results from sequencing batch tests showed that Na_2_SO_4_ influenced the decolourization efficiency more slightly than NaCl in different synthetic dye solutions with different mixtures of Na_2_SO_4_ and NaCl. In the dye solutions with the same salt concentration or the same Na^+^ concentration, high Na_2_SO_4_ concentration did not inhibit the decolourization process and even stimulated the decolourization efficiency of reactive brilliant red K-2BP. Compared to NaCl system, the addition of Na_2_SO_4_ increased the ORP values about 35 mV, which agreed with the theoretic analysis of Gibbs function. This study improved our knowledge of azo dye decolourization under high salinity conditions and provided efficient option for the treatment of azo dye wastewater.

## Introduction

Hyper-saline wastewater can cause plasmolysis and/or loss of activity of cells. Activated sludge is usually protected against high salinity by pre-treatment of the influent wastewater, and traditional aerobic and anaerobic biological processes used to treat saline water would lead to low biological oxygen demand removal performance.[1–3] Azo dyes are a class of dyes with one or more ‘-N = N-’ groups in their chemical structure and are used widely in textile fibres, plastics, leather, paper, mineral oils, waxes and cosmetics. Large amounts of inorganic salts, such as NaCl, Na_2_SO_4_ and NaNO_3_, are used in these dye manufacturing industries and dye-consuming industries, [4] and NaOH is commonly used to adjust the pH to the alkaline range, which leads to high salinity dye wastewater.[5] For example, Na_2_SO_4_ is a conventional additive of dye baths or is formed by the oxidation of more reduced sulphur species used in the dyeing process, such as sulphide, hydrosulphite and dithionite. Na_2_SO_4_ is also produced in the neutralization of alkaline dye effluents with sulphuric acid. The salt concentrations in some dyestuff industry wastewaters have been found to be as much as 25%–30%.[6] Today, azo dye decolourization is accomplished mainly by biological processes, and many azo dye decolourizing and salt-tolerant microorganisms have been reported in the past decades.[7,8] Previous studies mainly focused on how the high concentration of NaCl affected the dye biodecolourization.[9,10] However, few studies reported about the effect of Na_2_SO_4_ or mixture of NaCl and Na_2_SO_4_ on the biodecolourization process. That is why, the aim of this study was to investigate the effect of high concentrations of NaCl and/or Na_2_SO_4_ on the decolourization efficiency, and to explore how Na_2_SO_4_ affects the K-2BP decolourization by comparison with the changes of the oxidation–reduction potentials (ORPs) in the decolourization process and ORP_theoretic_ deduced from Gibbs function.

## Materials and methods

### Dye and reagents

The azo dye of reactive brilliant red K-2BP used in this study was from the School of Textile and Fashion, Hebei University of Science and Technology (Shijiazhuang, China). The chemical structure of the dye is shown in the online Appendix (Figure S1). All other reagents were analytical grade and were purchased from Xiandai Ltd (Shijiazhuang, China).

### Medium and culture conditions

The salt tolerant medium (STM) used for the decolourization has been previously described.[2] The STM composition was: 5 g·L^−1^ yeast extract, 10 g·L^−1^ peptone and 0–300 g·L^−1^ NaCl or 0 g·L–300 g·L^−1^ Na_2_SO_4_ (pH 7.0). The pH was adjusted with 1 mol L^−1^ NaOH or 1 mol L^−1^ HNO_3_.

Strain GYW was incubated aerobically in STM at 30 °C and pH 7.0 on a rotary shaker at 150 r·min^−1^. Then the cells in exponential growth were inoculated into 100 mL of STM containing designated dye concentrations and different NaCl and/or Na_2_SO_4_ concentrations. The initial cell concentration was approximately 0.39 g dry weight per litre in all the experiments.

### Identification and characterization of strain GYW

Strain GYW was originally isolated from aerobic wastewater sludge samples from the treatment plant of a local dyeing factory and cultivated in an enrichment culture medium with 50 mg·L^−1^ azo dye K-2BP as the indicators of microbial activity.

Genomic DNA was extracted from the cells by the described method.[11] The universal primers 8f and 1522r were used and the polymerase chain reaction (PCR) amplification of 16S rDNA was conducted in a total volume of 50 μL, containing 1 μL of 10 μmol·L^−1^ (each) primer, 4 μL of 2.5 mmol·L^−1^ (each) dNTP, 5 μL 10 × LA-taq buffer (20 mmol·L^−1^ Tris-HCl, 100 mmol·L^−1^ KCl, 25 mmol·L^−1^ MgCl_2_) and 0.3 μL of 5 U·μL^−1^ LA taq (TaKaRa, Dalian Co., Ltd). The PCR product was purified by using a TaKaRa quick PCR purification kit (TaKaRa, Dalian Co., Ltd). The purified 16S rDNA was then sequenced using TaKaRa, Dalian Co., Ltd. The 16S rDNA sequence of strain GYW was aligned with 16S rRNA gene sequences of *Halomonas* species in the Genbank database by using the BLAST program.

To characterize the morphological and physiological characteristics of the isolate, cell morphology was examined by transmission electron microscopy. Standard phenotypic tests were performed, including Gram reaction, cell morphology, motility, growth under anaerobic conditions, catalase and oxidase production.

### Effect of different environmental factors on the decolourization efficiency

Batch tests were all performed in 125 mL serum bottles with rubber stopper. The anaerobic decolourization of azo dye by strain GYW was carried out in Dye-STM with 100 mg·L^−1^ reactive brilliant red K-2BP, under different salt concentrations (0%–30%), different temperatures (20–40 °C) and different pH (5.0–10.0) conditions. To prevent possible contamination by oxygen during sampling, bottles were opened only once, and as many bottles were incubated as measurements were planned. Controls with cell-free and autoclaved bacteria were incubated. All tests were conducted in triplicate to check the accuracy of the results throughout the dye decolourization experiments.

### Effect of NaCl and/or Na_2_SO_4_ on the decolourization under different saline conditions

NaCl and Na_2_SO_4_ are common inorganic salts found in textile wastewaters. To investigate the effect of NaCl and/or Na_2_SO_4_ on the biological decolourization of K-2BP by strain GYW, batch experiments with different mixtures of Na_2_SO_4_ and NaCl were performed. There were two experiment runs: in the first one, the total salt concentration was kept constant at 10% with different content of NaCl and/or Na_2_SO_4_; in the second one, the concentration of Na^+^ was kept constant with different NaCl and/or Na_2_SO_4_ concentrations. The assays were performed in triplicate, with a control without bacteria.

### ORP change during the decolourization process under different saline conditions

ORP is also an important parameter for the effective decolourization of azo dyes, but there are few studies on the effect of inorganic salts, especially sulphate, on the ORP change during the decolourization process. To study the relation between ORP and NaCl/Na_2_SO_4_ in the biodecolourization process, two experiments with the same salt concentration and the same Na^+^ concentration of dye solutions were carried out. The ORP changes were measured during the biodecolourization process. The assays were performed in triplicate.

### Analytical methods

The cell concentration was measured by optical density at 660 nm and converted to dry cell weight [OD_660_ of 0.720 = l g·L^−1^, (*R* ^2^ = 0.997)]. The pH was determined with a digital pH Meter (Delta-320, China). ORP was measured with a digital pH Meter (Delta-320, China) and an ORP composited electrode (Leici-501, China). Sulphide was measured by ion chromatography (Metrohm IC-761, Switzerland). Samples previously centrifuged (8000*g* for 10 min) and filtered (0.45 μm Millipore) were separated and eluted using a Metrosep A Supp 4-250 analytical column with carbonate/bicarbonate/acetone eluent (1.8 mmol·L^−1^ Na_2_CO_3_/1.7 mmol·L^−1^ NaHCO_3_/100 mL L^−1^ acetone at 1 mL·min^−1^).

Samples were taken at regular intervals and centrifuged at 8000*g* for 10 min (Sigma, 3-16PK, Germany). The supernatants were then used for the determination of decolourization efficiency. To determine the concentration of reactive brilliant red K-2BP, absorbance of the sample was measured at a wavelength of 538 nm (λ_max_) on an ultraviolet-visible spectrophotometer (Tianmei, UV-2600, China), and absorbance was proportional to concentration in the test range. The decolourization efficiency of K-2BP (E) was obtained as follows:(1) 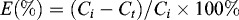
where *C_i_* and *C_t_* are the initial and residual dye concentration, respectively.

## Results and discussion

### Identification and characterization of strain GYW

The isolated strain GYW was identified as a member of the genus *Halomonas* according to its physiological–biochemical characteristics and 16S rRNA gene sequence. The Gene Bank accession number of strain GYW is EF188281.

It is a Gram-negative, non-motile, rod-shaped, facultative bacterium, 0.6–1.0 μm wide and 1.0–2.0 μm long. No flagellum was found by electron microscopy. The transmission electron micrograph of strain GYW is shown in the online Appendix (Figure S2). Colonies were mucoid, circular, low convex and yellow. It was found to be oxidase positive, catalase positive and denitrification negative. Acid could be produced from hydrolysis of glucose and starch.

### Effect of different environmental factors on the decolourization efficiency

Many factors, such as temperature, pH and mineral salt concentration, were reported to affect the decolourization efficiency by microorganisms.[12] In sterile controls, only a 0.2% change in the dye concentration over 48 h was observed (data not shown). The effects of temperature and pH on the decolourization efficiency are shown in the online Appendix (Figure S3). The results suggested that the optimal temperature and the appropriate pH range were 30 and 35 °C and 7.0–8.0, respectively.

**Figure 1.**
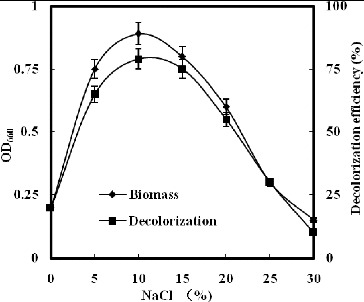
Effect of NaCl on growth and decolourization of K-2BP by strain GYW.

The results in Figure 1 show that strain GYW did not grow well outside the range of 5%–15% NaCl, and exhibited a maximum growth rate at 10% NaCl. This indicates that strain GYW is a halo-tolerant organism that is capable of growing in a saline environment and strictly requires NaCl for its growth.

### Effects of NaCl and/or Na_2_SO_4_ on the decolourization efficiency

**Figure 2.**
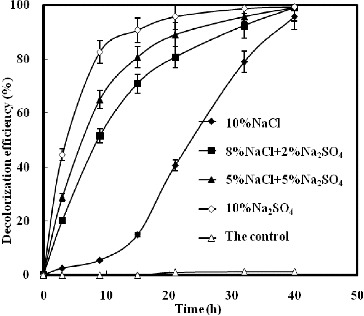
Decolourization of K-2BP by strain GYW at 10% salt concentration.

**Figure 3.**
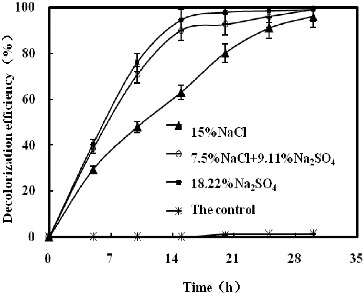
Decolourization of K-2BP by strain GYW at the same sodium ion concentration.

Figure 2 illustrates the effect of NaCl and/or Na_2_SO_4_ on K-2BP decolourization by strain GYW under 10% salt concentration. The results showed that the decolourization efficiency with NaCl only reached 14.9% after 15 h; however, a sharp increase was observed in the other studied variants. The decolourization efficiency increased proportionally to the concentration of Na_2_SO_4_ under constant salt concentration (10%), and Na_2_SO_4_ influenced the decolourization efficiency more slightly than NaCl. The decolourization efficiency with Na_2_SO_4_ only could reach 90.7% after 15 h. Figure 3 demonstrated the effect of NaCl and/or Na_2_SO_4_ on decolourization of K-2BP by strain GYW at the same sodium ion concentration. Up to 94.3% K-2BP could be degraded in 15 h in the presence of 18.22% Na_2_SO_4_, whereas only 62.9% K-2BP was degraded in 15 h in the presence of 15% NaCl. These results suggested that Na_2_SO_4_ could increase the decolourization efficiency of azo dye at the same cation concentration, which might be explained by the chemical reduction of H_2_S.[13]

### ORP change during the decolourization process

**Figure 4.**
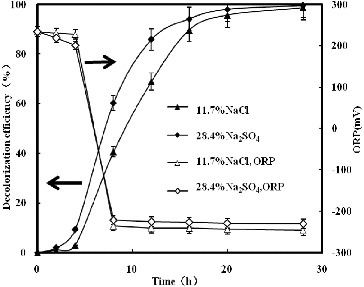
Decolourization of K-2BP and ORP profiles at the same anionic concentration.

**Figure 5.**
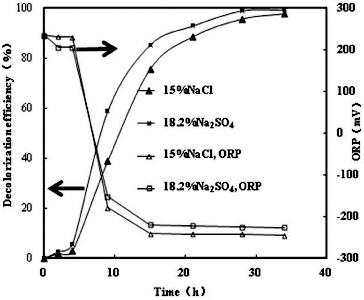
Decolourization of K-2BP and ORP profiles at the same sodium ion concentration.

Under no addition of sulphate, the ORP values decreased from an initial value of 230–235 mV and stabilized around −250 mV in anoxic conditions (Figures 4 and 5). During the aerobic incubation, only 1% absorbance decline at 538 nm was observed. However, rapid decolourization was observed when the ORP values dropped below ca. −200 mV under anoxic conditions, and the decolourization efficiency at 20 h was 95.7%. Similar results have also been reported by authors.[1] Figures 4 and 5 show similar ORP changes during the decolourization process by strain GYW. The ORP value, however, stabilized around −215 to −218 mV after 8 and 15 h anoxic conditions, respectively, which might be caused by the combination of the sulphate reduction and the decolourization. Sulphate can readily be reduced to sulphide by strain GYW. The biogenic sulphide could be involved in the decolourization process, because it might play a reductive role for azo decolourization. Therefore, sulphate increased the ORP value around 35 mV. The sulphide form was detected during the decolourization processes. The acceleration effect did not agree with other similar results,[14] which might be due to different microorganisms used and different mechanisms of decolourization.

### Theoretical analysis of the ORP change during the decolourization process

Theoretically, biological decolourization of azo dyes is a non-specific and presumably extracellular process, which includes three different mechanisms: direct enzymatic reduction, indirect/mediated reduction and chemical reduction.[15–18] According to this mechanism, the process of biological decolourization is an oxidation–reduction reaction, in which the electron transfer matches with the proton flow by the help of NADPH/NADP^+^ and NADH/NAD^+^. The ORPs of the pairs NADPH/NADP^+^ and NADH/NAD^+^ are −324 and −320 mV, respectively.[19,20] The least 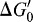 value of the conversion NADPH/NADP^+^ and NADH/NAD^+^ is 44 kJ.[21] Therefore, −93 mV, which was obtained from Equations (2) (Gibbs function) and (3), could be considered as a rough limited ORP value for ordinary primary electron donors of the mechanism of biological azo dye reduction. This was demonstrated by the experimental results (Figures 4 and 5).(2) 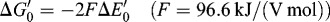

(3) 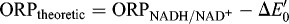
However, the biogenic sulphide was a reducer for the decolourization process instead of NADPH/NADP^+^ and NADH/NAD^+^. The highest ORP_theoretic_ of the decolourization process with sulphide was enhanced 42 mV, which was obtained from Equations (2) and (4), and could be considered as a rough limited ORP value for ordinary primary electron donors of the biological reduction mechanism. The ORP of the pair H_2_S/S^2−^ is −270 mV, which is higher than that of NADH/NAD^+^, therefore sulphate increased the ORP values during the decolourization process. The comparison showed that the enhanced ORP values (35 mV) measured in the experiment agreed with the theoretically enhanced ORP values (42 mV). This suggested further that sulphide played a reductive role in the decolourization process.(4) 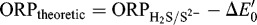



The results indicate that biodecolourization of azo dyes is significantly affected by inorganic salts, and there is a close relationship between the ORP and decolourization efficiency. This study could provide a theoretical foundation for practical application of high salinity dye wastewater treatment.

## Conclusions

In the present study, *Halomonas* sp. GYW was isolated and characterized. Its optimal temperature, pH and the appropriate NaCl concentration range were 30–35 °C, 7.0–8.0 and 5%–15%, respectively. During the azo dye decolourization process, high sulphate could enhance the decolourization efficiency of K-2BP. This might be attributed to sulphide, which was reduced from sulphate by strain GYW and played a reductive role in the decolourization process. Azo dyes were reduced by strain GYW when the ORP values decreased to certain limits (<−93 mV), and stabilized between −215 and −250 mV during the decolourization process. Sulphate could increase the ORP values around 35 mV with the theoretical analysis of Gibbs function. This study could provide a theoretical foundation for practical application of high-salinity dye wastewater treatment.

## Supplementary Material

Supplementary Appendix
