# Supplementary material for: Effect of NaCl and Na2SO4 on the biodecolourization of K-2BP by Halomonas sp. GYW
Source: Biotechnol Biotechnol Equip. 2014 Apr 30;28(1):38–42. doi: 10.1080/13102818.2014.901677 (PMC4987067; doi:10.1080/13102818.2014.901677)
Supplement: Supplementary Appendix [file tbeq_a_901677_sm9165.doc]

**SUPPLEMENTARY APPENDIX**

**DOI:** 10.1080/13102818.2014.901677

This appendix has been provided by the authors to give readers additional information about their work.

Supplement to:

**EFFECT OF NaCl AND Na_2_SO_4_ ON THE BIODECOLORIZATION OF K-2BP BY *HALOMONAS SP*. GYW**

Jing Lian, Zhifang Xu, Jianbo Guo^*^, Lin Yue, Yankai Guo, Chenxiao Zhang, Jingliang Yang

Biotechnol. & Biotechnol. Eq. 2014, DOI: 10.1080/13102818.2014.901677.

**Supplementary Appendix**

**Fig. S1.** Chemical structure of the azo dye K-2BP.


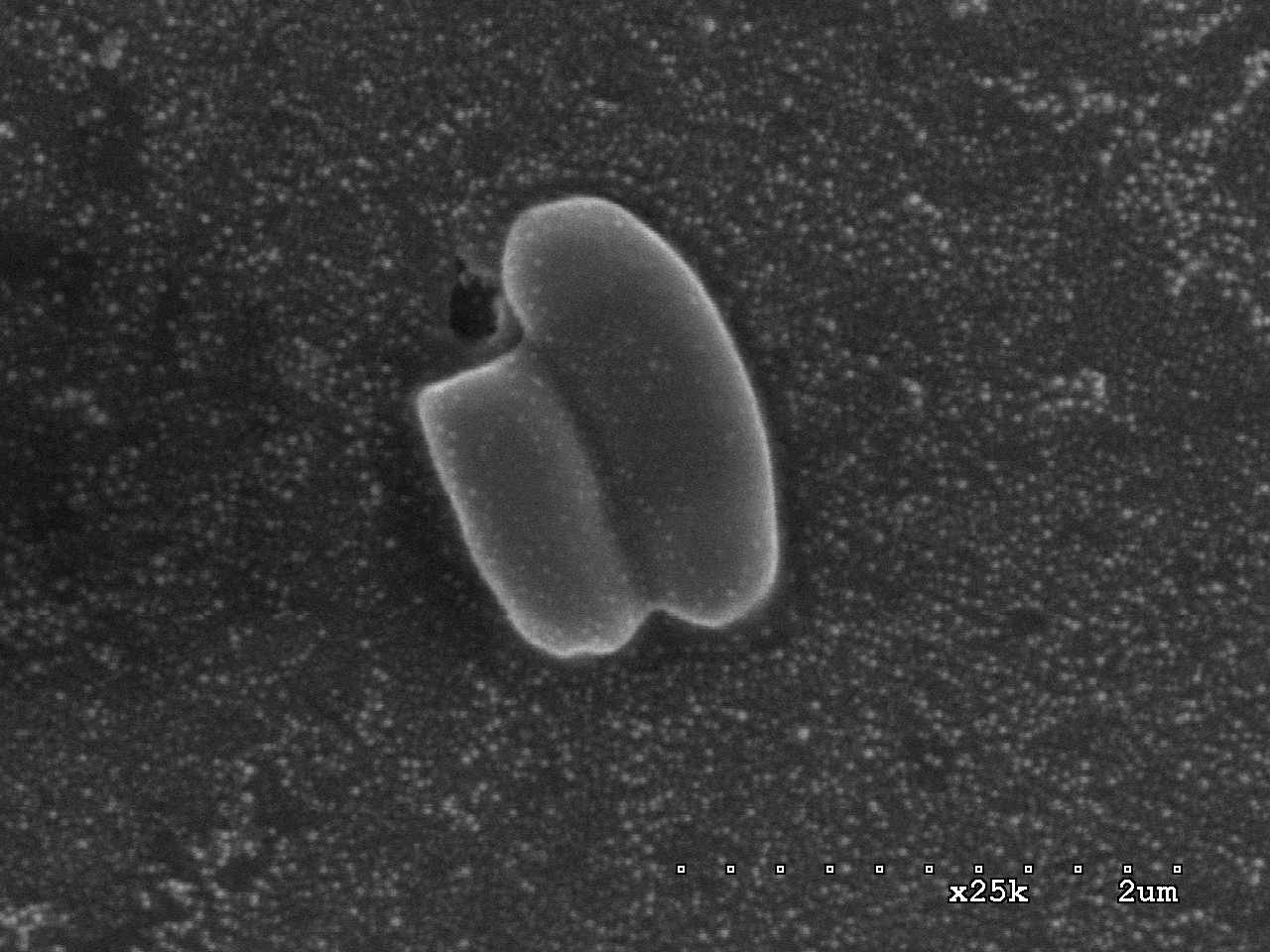

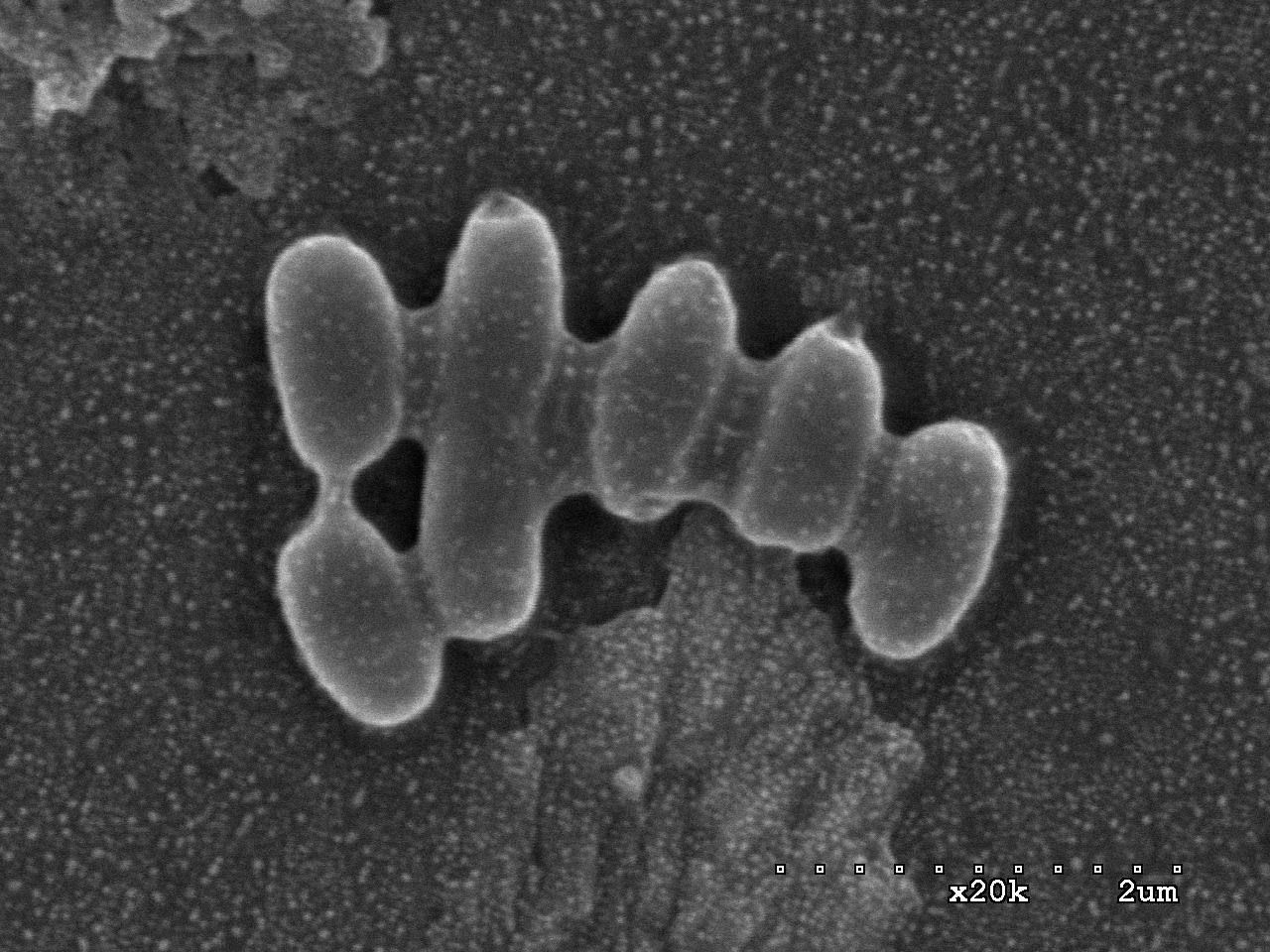


**Fig. S2** Transmission electron micrograph of strain GYW.

**A**
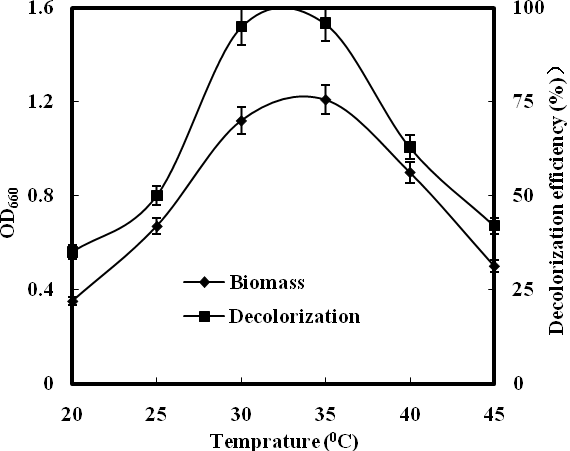
**B**
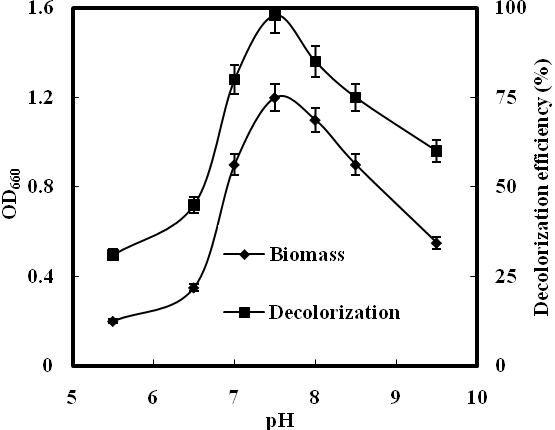


**Fig. S3.** Effect of temperature (**A**) and pH (**B**) on the growth of strain GYW and the decolorization of K-2BP.
